# The Effective Ropivacaine Concentration for Transversus Thoracic Muscle Plane Block in Adult Cardiac Surgery: A Dose-Finding Trial Protocol

**DOI:** 10.7759/cureus.97901

**Published:** 2025-11-26

**Authors:** Mayuko Onishi, Keitaro Tachi, Kazushi Maruo, Bryan Mathis, Yosuke Nakadate

**Affiliations:** 1 Department of Anesthesiology, University of Tsukuba Hospital, Tsukuba, JPN; 2 Department of Biostatistics, Institute of Medicine, University of Tsukuba, Tsukuba, JPN; 3 Department of Cardiovascular Surgery, Institute of Medicine, University of Tsukuba, Tsukuba, JPN; 4 Department of Anesthesiology, Institute of Medicine, University of Tsukuba, Tsukuba, JPN

**Keywords:** biased-coin up-and-down method, cardiac surgery, dose-finding trial, ropivacaine, transversus thoracic muscle plane block

## Abstract

Background

Median sternotomy in cardiac surgery is associated with severe pain, and opioid-based analgesia is currently standard. In recent years, various ultrasound-guided nerve block types have been used in cardiac surgery to reduce opioid use and facilitate postoperative recovery, including ultrasound-guided transversus thoracic muscle plane block. However, the 90% effective concentration of ropivacaine used for ultrasound-guided transverse thoracic muscle plane block (TTPB) in cardiac surgery remains undetermined. This study aims to evaluate the 90% effective concentration of ropivacaine for ultrasound-guided transversus thoracic muscle plane block in adult cardiac surgery using the biased coin up-and-down sequential method.

Methods

This study is a single-center, prospective, sequential allocation dose-finding trial. Participants will be adult patients with American Society of Anesthesiologists physical status (ASA-PS) II-IV undergoing elective cardiac surgery with median sternotomy. Ultrasound-guided transversus thoracic muscle plane block will be performed after induction of general anesthesia at 0.4 ml.kg^-1^ ropivacaine per side (0.8 ml.kg^-1^ bilaterally), with concentrations pre-determined by biased coin up-and-down sequential method. Successful transversus thoracic muscle plane block is to be defined as less than a 20% increase in heart rate or blood pressure from the baseline value within one minute after the skin incision in the midline of the chest. The 90% effective concentration of ropivacaine will be estimated using isotonic regression.

Conclusions

The results of this study will help establish guidelines for transversus thoracic muscle plane block in cardiac surgery and may facilitate the optimization of healthcare resources.

## Introduction

Background and rationale

Good perioperative pain management affects patient recovery and quality of life, especially in cardiac surgery. Median sternotomy is known to be extremely painful and systemic administration of opioids has traditionally been used for perioperative analgesia [1,2]. However, this method carries side effects, such as severe constipation, nausea and vomiting, respiratory depression, prolonged intubation periods, and hyperalgesia [3]. Insufficient management can lead to chronic pain at the wound site, which reduces the patient’s postoperative outcome and quality of life [1,2]. Therefore, multimodal perioperative analgesia, including non-narcotic analgesics and regional anesthesia, is important. Among regional anesthesia, neuraxial anesthesia (such as epidural anesthesia) is also an effective option for perioperative analgesia after thoracic surgery, but in cardiac surgery, the increased risk of epidural hematoma due to systemic heparin administration during surgery is a concern [2,4].

In recent years, with the improvement of ultrasound-guided regional anesthesia techniques, various nerve blocks have been used for perioperative pain management following cardiac surgery [5-8]. Ultrasound-guided transverse thoracic muscle plane block (TTPB) is an ultrasound-guided nerve block that was first reported in 2015 [9,10]. In TTPB, local anesthetic is injected into the fascial plane between the transverse thoracic muscle and the internal intercostal muscles to provide analgesia in the anterior cutaneous branch region of the intercostal nerves (T2-6) in the anterior chest, making it effective for wound pain following median sternotomy and reducing the need for opioids [9-12]. Other chest wall blocks reported for cardiac surgery include the pectoral nerve (PECS) block and the parasternal intercostal block (PIPB) [5,13]. The analgesic coverage of the PECS block is limited to the lateral chest wall and is not suitable for analgesia during median sternotomy [5]. The PIPB targets the anterior branches of the intercostal nerves by injecting local anesthetic into the fascia between the pectoralis major and external intercostal muscles, making it effective for median sternotomy analgesia [13]. However, it may provide inadequate blockage of the medial branches of the intercostal nerves and requires puncture of multiple intercostal spaces [13]. In contrast, unlike PIPB, TTPB can cover analgesia for median sternotomy incisions with a single intercostal puncture [13]. However, reports of TTPB as an analgesic strategy for adult cardiac surgery featuring median sternotomy do not currently feature guidelines on establishing the ideal concentration and dose of ropivacaine.

Objectives

The primary objective of this study is to determine the concentration of ropivacaine that provides effective analgesia in 90% of adult cardiac surgery patients (90% effective concentration: EC90) undergoing median sternotomy with ultrasound-guided TTPB. The effectiveness of TTPB at the start of surgery will be evaluated using the change in hemodynamics during skin incision as an index of noxious stimulation.

Trial design

This single-center, prospective, interventional, sequential allocation dose-finding study, using the biased coin up-and-down design targeting EC90, is designed to determine the optimal concentration of ropivacaine for ultrasound-guided TTPB during adult cardiac surgery.

## Materials and methods

Study setting

The trial will be conducted by the Department of Anesthesiology within the University of Tsukuba Hospital.

Eligibility criteria

The following inclusion (Table 1) and exclusion criteria (Table 2) will be applied to all potential enrollees.

**Table 1:** Inclusion criteria.

Inclusion criteria
Patients undergoing cardiac surgery via median sternotomy
ASA-PS (American Society of Anaesthesiologists physical status classification) II - IV
Patients aged 18 to 80 years at the time of informed consent

**Table 2:** Exclusion criteria.

Exclusion criteria
History of median sternotomy
Malformation, nerve damage or infection near the nerve block puncture site
History of allergies to local anaesthetics or drugs used for general anaesthesia
Pregnant
New York Heart Association functional classification Class IV symptomatic heart failure
Pulmonary hypertension requiring inhaled nitric oxide
Preoperative artificial ventilation management
Severe blood coagulation disorder
A serious systemic illness
Cases of infective endocarditis
Patients taking drugs that inhibit cytochrome P450 1A2 (CYP1A2)
Patients who are deemed inappropriate by the principal investigator (or sub-investigator)

Intervention description

General anesthesia will be induced as required for adult cardiac surgery. Ultrasound-guided TTPB is to be performed after oral tracheal intubation. Before ultrasound-guided TTPB, the anatomy of the puncture site (bilateral parasternal region) will be confirmed using a linear probe with an effective frequency range of 4-18 MHz and a center frequency of 10 MHz (L18-4, Konica Minolta, Tokyo, Japan) on an ultrasound device (SONIMAGE HS2, Konica Minolta, Tokyo, Japan). For ultrasound-guided TTPB, the ultrasound probe is to be placed parallel to the ribs at the 4th/5th intercostal space 1 cm lateral to the sternal edge, and the internal intercostal muscles, transverse thoracic muscles, and pleura must be clearly identified before puncture is then performed using a 22G block needle in the parallel technique. After confirming that the block needle has advanced to the transverse thoracic fascia surface, 3 ml of saline will be test-injected per side to confirm hydro-dissection of the transverse thoracic fascia surface. Then, ropivacaine (Anapeine, Sandoz Pharma K.K., Tokyo, Japan) will be administered at 0.4 ml.kg^-1^ on one side (0.8 ml.kg^-1^ on both sides). The concentration of ropivacaine used in TTPB is to be determined in advance using a biased coin designed up-and-down method (BC-UDM) [14-16]. The maximum dose of ropivacaine was set at 300 mg in accordance with the package insert for Japanese ropivacaine formulations. Based on this, the maximum tolerated dose of ropivacaine in BC-UDM was set at 3.2 mg.kg^-1^ (ropivacaine concentration 0.4%). An experienced anesthesiologist will administer the above treatment. The image displayed on the ultrasound device during injection will be printed out, and an image evaluator can then judge whether the local anesthetic has been properly injected into the transverse thoracic fascia. After TTPB, a central venous catheter and (if necessary) a pulmonary artery catheter are to be placed. A post-scan will then be performed using an ultrasound device in the parasternal region to confirm that the local anesthetic has spread to the transverse thoracic fascia surface in the parasternal region in the T2-6 range. After the block, patients will be monitored with electrocardiography and invasive arterial pressure measurement. A 20% fat emulsion will be kept readily available in the operating room. In the event of systemic toxicity, lipid therapy will be initiated with a 1.5 mL/kg bolus of 20% fat emulsion, repeated as necessary, followed by a continuous infusion at 0.25 mL/kg/min. In cases of severe circulatory instability, extracorporeal circulation will be established immediately in cooperation with the cardiac surgery team. Surgery will be started at least 20 minutes after TTPB, and the effectiveness of TTPB will be evaluated when making an incision in the skin in the midline of the chest.

Permitted care

General anesthesia will be induced by intravenous injection of midazolam, remimazolam, or propofol, and inhalation of 1-5% sevoflurane. After muscle relaxation is achieved by intravenous injection of 0.6-1.0 mg.kg^-1^ rocuronium, remifentanil will be administered continuously at 0.2-0.4 µg.kg^-1^.min^-1^ for about 5 minutes, followed by oral tracheal intubation. The maintenance concentration of sevoflurane after tracheal intubation will be adjusted to an exhaled concentration of 1-1.5%. The continuous administration of remifentanil will be discontinued after disinfection of the surgical field, and no additional administration is to be given until median sternotomy. Before the start of surgery, the state of muscle relaxation will be evaluated using a train-of-four (TOF) monitor. If the TOF is 1 or higher, an additional 10-20 mg of rocuronium is to be administered. The effectiveness of TTPB will be assessed during the period from the skin incision at the start of surgery to the median sternotomy. Sympathetic nervous responses (heart rate and blood pressure) were evaluated as indicators of noxious stimulation. If the blood pressure or heart rate increases by 20% or more from the baseline value measured at the end of the induction of anesthesia within 1 minute after the skin incision in the midline of the chest, it will be judged as failed TTPB and, if the increase is less than 20%, it will be judged as successful TTPB [17-20]. If the ultrasound-guided TTPB block is unsuccessful, a bolus of 0.1-0.2 µg.kg^-1^ of remifentanil will be administered immediately as a rescue. After efficacy assessment of TTPB, there are no restrictions on remifentanil or fentanyl and any administration will occur at the discretion of the attending anesthesiologist. After intubation, the ventilator settings will be set to FIO2 0.4, and the inspiratory pressure and ventilation rate will be adjusted in pressure-controlled mode to achieve an exhaled carbon dioxide of 35-40 mmHg.

Prohibited care

Ropivacaine is metabolized by the hepatic enzyme CYP1A2. Because this may potentially affect the results of the study, patients using the drugs that have a CYP1A2 inhibitory effect (vemurafenib and anastrozole used within four weeks prior to surgery, and other drugs used within one week prior to surgery) will be excluded from the study, and use of such concomitant drugs is not permitted under this protocol (Table 3).

**Table 3:** List of prohibited drugs during this trial.

Common name	Drug Category Name	Japanese Product Name
Famotidine	H2 receptor antagonists	Gaster
Ticlopidine	Antiplatelet agents	Panaldine
Ciprofloxacin	New quinolone antibacterial agents	Ciproxan
Norfloxacin	New quinolone antibacterial agents	Baccidal
Pazufloxacin	New quinolone antibacterial agents	Pasil, Pazucross
Vemurafenib	Anticancer drugs (BRAF inhibitors)	Zelboraf
Anastrozole	Aromatase inhibitors for postmenopausal breast cancer treatment	Arimidex
Allopurinol	Hyperuricemia treatment	Zyloric
Stiripentol	Antiepileptic drugs	Diacomit
Fluvoxamine Maleate	Selective serotonin reuptake inhibitors (SSRIs)	Depromel, Lubox
Disulfiram	Anti-alcoholism drugs	Nocbin
Methylthioninium chloride hydrate	Treatment for methemoglobinemia	Methylene blue
Fomepizole	Ethylene glycol/methanol poisoning agent	Fomepizole
Deferasirox	Iron chelators	Jadenu
Ritrecitinib Tosilate	Disease-modifying antirheumatic drugs (Janus kinase inhibitors)	Litfulo

Outcomes

Primary Outcome

The primary outcome of this study is the EC90 of ropivacaine for use in ultrasound-guided TTPB, calculated from the success or failure of ultrasound-guided TTPB at the start of cardiac surgery via median sternotomy. Changes in heart rate and blood pressure were used to assess nociceptive responses. A ≥20% increase from the baseline value within 1 minute after the midline chest incision indicated an unsuccessful TTPB, while a <20% increase indicated success [17-20].

Secondary Outcomes

The need for additional narcotics (fentanyl, remifentanil) between skin incision at the start of surgery and median sternotomy will be evaluated.

Harms

Anticipated adverse events include: 1) Local anesthetic toxicity: Severe local anesthetic toxicity of convulsions or worse occurs at a frequency of 2.6/10,000 with ultrasound-guided nerve blocks [21]. 2) Tissue damage due to puncture: pneumothorax (frequency of occurrence is 4/10,000 with ultrasound-guided nerve blocks) [21,22], vascular damage, cardiac damage. 3) Nerve damage due to puncture (frequency of occurrence is 4/10,000 with ultrasound-guided nerve blocks) [21,23]. 4) Infection at the puncture site.

Complications due to the puncture are assessed by post-procedure scanning with an ultrasound device and observation in the surgical field. Complications due to drug administration are assessed based on changes in vital signs. The content, extent, treatment, outcome, and severity of the complications are recorded in the medical record and case report, and follow-up surveys are conducted if necessary (those due to the administration of general anesthesia are not covered). If an adverse event occurs, medically necessary treatment and care will be provided under the guidelines of national health insurance, even after the completion of the trial.

Participant timeline

The timeline of participants is shown in the table below (Table 4).

**Table 4:** The participant timeline. X indicates timing of enrolment, intervention, and assessments. - indicates not applicable.

	Screening Period	Study Period				
Time point	From surgery decision to the day before surgery	On the day of surgery				The day after surgery
		Anesthesia induction	Skin incision	Median sternotomy	End of surgery	
Obtaining consent	X	-	-	-	-	-
Eligibility screen	X	-	-	-	-	-
Registration	X	-	-	-	-	-
Check vital signs	X	X	X	X	X	X
Ultrasound pre-scan of the puncture site	-	X	-	-	-	-
Intervention (transversus thoracic muscle plane block)	-	X	-	-	-	-
Ultrasound post-scan of the puncture site	-	X	-	-	-	-
Check for muscle relaxation	-	X	-	-	-	-
Primary outcome	-	-	X	-	-	-
Secondary outcome	-	-	-	X	-	-
Check for postoperative surgical site infection	-	-	-	-	-	X
Check for postoperative pneumonia	-	-	-	-	-	X
Safety assessment	X	X	X	X	X	X

Sample size

A stopping rule is necessary in dose-finding studies based on a BC-UDM [15]. A minimum sample size of n = 50-60 is recommended for calculating EC90 [14]. The stopping rule is set at 45 successful blocks [16]. The target sample size is thus 70 cases, with an estimated dropout rate of 10%.

Recruitment

Prior to the day of surgery, researchers will review the medical records of patients scheduled for surgery and determine potential participants based on the inclusion and exclusion criteria. Researchers will then visit the patients and ask them to participate in the study.

Assignment of interventions: allocation

**Figure 1:**
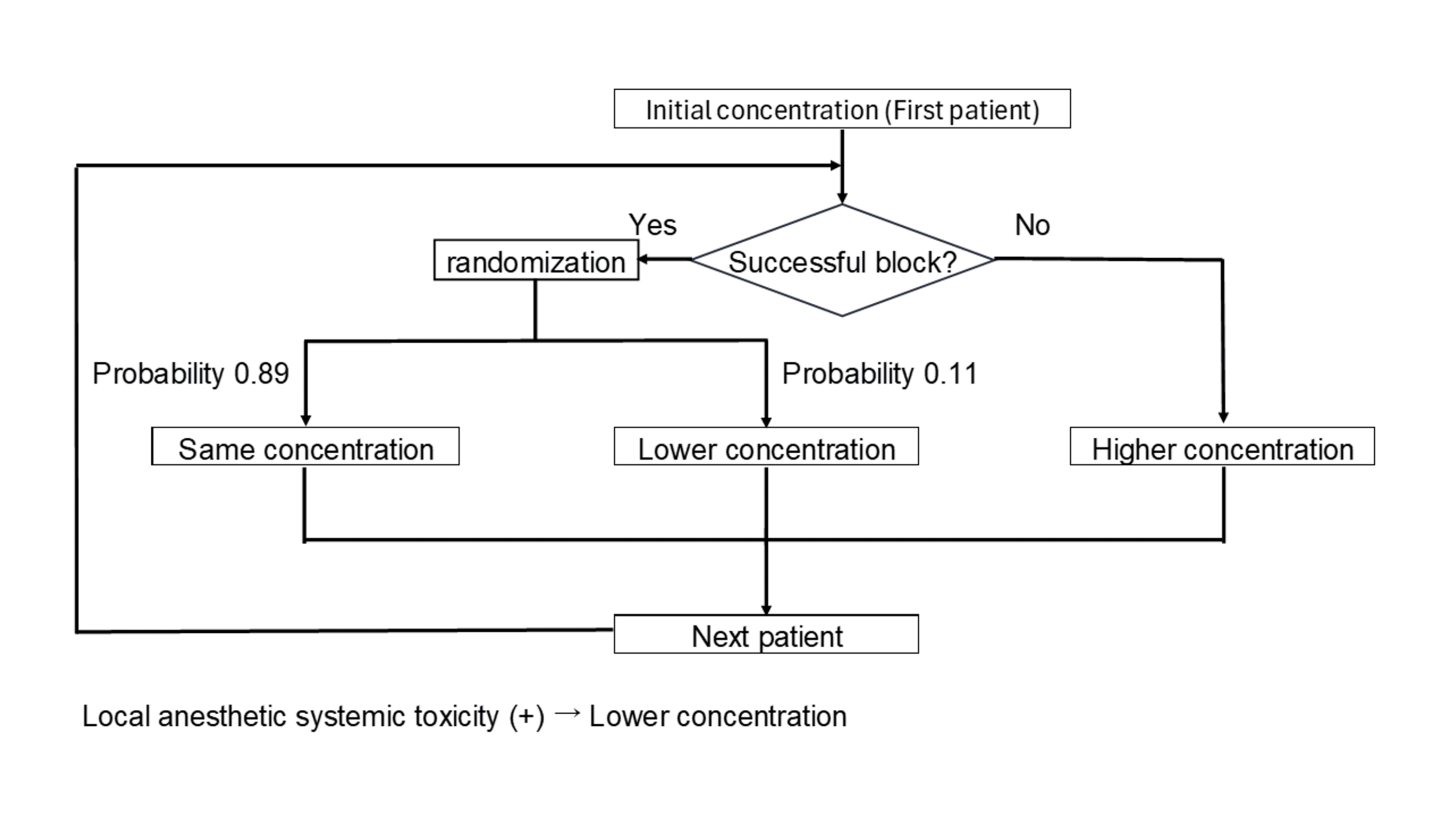
Flow chart of allocation procedure.

In the first case, 0.2% ropivacaine is used. In subsequent cases, the concentration of ropivacaine is to be determined based on the success or failure of the previous block according to a BC-UDM (Figure 1) [14-16]. For the calculation of EC90, if the previous block was unsuccessful, the next higher concentration (increased or decreased by 0.025%) is to be used, and if the block was successful, there is an 8/9 chance of using the same concentration and a 1/9 chance of using the next lower concentration. The floor dose of ropivacaine is 0.1% and the ceiling dose is 0.4%. If the floor dose of 0.1% ropivacaine is successful, the next patient will be assigned to the same concentration (0.1%) and, if the ceiling dose of 0.4% ropivacaine is unsuccessful, the next patient will be assigned to the same concentration (0.4%). Randomization of successful blocks is to be performed using computer-generated random numbers. The patients are to be registered after obtaining consent. The biostatistician of the study will create the random allocation sequence and communicate it by email to the researcher (YN) in charge of allocation. YN will then enroll and allocate participants.

Blinding

The researcher who will not perform TTPB is informed of the allocation and will have the local anesthetic prepared outside the operating room before the patient enters the room. The concentration of the local anesthetic is not to be written on the syringe so that study participants and the researcher who will perform TTPB (KT) will be blinded to the ropivacaine concentration.

The researcher performing the TTPB and blinded to the concentration of ropivacaine (KT) will assess whether the TTPB is successful or not, and will collect data at the start of surgery. Another member of the research group who is not blinded to the concentration of ropivacaine (MO) will collect the remaining perioperative data.

Data collection

Research data for this study will be collected from electronic medical record systems or case report forms (CRFs). Preoperative data, including demographics, medical history, medication history, laboratory test results, and preoperative evaluations, will be completed on a CRF by the investigator prior to surgery.

Data management

All data will be anonymized and CRFs and electronic data will be coded with a subject identification code. Electronic data recording media and paper documents will be stored on lockable shelves in a lockable room and kept locked at all times. Deleted information, etc., will be stored in a file/folder separate from the analysis data with a password and managed by the principal researcher. As this is personal information, it will be recorded as an electronic file using a computer that is not connected to the Internet and will be password-locked on recording media outside the computer.

Statistical methods

Primary Outcome

The EC90 is defined as the dose of ropivacaine at which successful block was observed in 90% of patients in the study population. EC90 will be calculated using isotonic regression [14-16]. The estimator to be used for isotonic regression is the adjusted success rate of block at each concentration, estimated by the pooled-adjacent-violators algorithm (PAVA) [15,16]. The 90% confidence interval for the isotonic regression estimate of EC90 (Û3) is to be calculated using the bias-corrected percentile method using 2000 bootstrap replicates of Û3 [15,16]. All statistical analyses will be conducted with R version 4.5.0 or later (R Core Team, Vienna, Austria).

Secondary Outcomes

Summary statistics will be calculated. Exploratory model analyses may be performed as needed.

## Results

Dissemination plans

This is an ongoing study protocol and the results are going to be collected and analysed in 2027. The results of this study will be presented at relevant scientific conferences and published in peer-reviewed journals. The datasets used and analysed during the current study are available from the Principal Investigator on reasonable request. Although patients and members of the public were not directly involved in the design or conduct of the trial, the Institutional Review Board reviewing this protocol includes patient and public representatives. These members contribute to the ethical oversight of the study by providing perspectives on participant burden, informed consent materials, and the relevance of study outcomes to the patient community.

## Discussion

This study was designed to investigate the effect of TTPB on wound pain at the start of surgery. To our knowledge, no studies have investigated the direct effect of fascial plane block on wound pain at the start of abdominal or thoracic surgery in adults. Therefore, the methodology of this study can be considered novel.

Ultrasound-guided fascial plane blocks are a method of injecting a local anesthetic between two fascial layers to block the nerves that innervate the intermuscular layers, providing analgesia over a relatively large area, such as the chest or abdominal walls. This type of regional anesthesia is relatively easy to perform and has a low risk of complications [24, 25]. Ultrasound-guided TTPB, a type of fascial plane block, is effective for treating wound pain following median sternotomy because it is localized at the fascial plane between the transverse thoracic muscle and the internal intercostal muscles to provide analgesia in the anterior cutaneous branch area of ​​the intercostal nerves (T2-6) in the anterior chest [9, 10]. Unlike paravertebral and erector spinae plane blocks, this technique retains the advantages of compatibility with the supine position and does not require positioning in high-risk patients. On the other hand, it has been reported that chest wall nerve blocks result in faster absorption of local anesthetics and are more likely to increase blood concentrations of local anesthetics than nerve blocks in other body areas [26]. As there is limited literature on the application of ultrasound-guided TTPB in cardiac surgery, reporting the appropriate dose of ropivacaine used for ultrasound-guided TTPB will contribute to patient safety by reducing the risk of local anesthetic toxicity while obtaining adequate analgesia.

Enhanced Recovery After Surgery (ERAS) guidelines for cardiac surgery have highlighted perioperative pain, prolonged mechanical ventilation, and bed rest as important factors for patients remaining hospitalized after cardiac surgery [27, 28]. Although new cardiac surgery approaches, such as minimally invasive cardiac surgery and robotic-assisted surgery, have emerged, median sternotomy is still used in cardiac surgery, but is known to be extremely painful [1,2]. Opioid-based “balanced anesthesia” has long been used in cardiac surgery to achieve good perioperative analgesia in cardiac surgery [2, 28]. However, considering side effects (e.g., nausea, vomiting, sedation, and/or respiratory depression) [3], opioid-based analgesia management for cardiac surgery needs to be reconsidered. Recently, multimodal pain management, including regional anesthesia, has become an important component of ERAS [2, 29]. The use of ultrasound-guided TTPB in combination with median sternotomy wound analgesia may help reduce opioid use [10-12] and opioid-related side effects, shorten ICU and hospital stays, contribute to the refinement of multimodal analgesia and ERAS protocols, improve the quality of postoperative rehabilitation, and promote the optimization of healthcare resource utilization. To clarify the EC90 of ropivacaine for TTPB will contribute to patient safety by reducing the risk of local anesthetic toxicity while obtaining adequate analgesia. The finding of an adequate ropivacaine dose will provide a benchmark for setting ropivacaine doses in future, randomized, controlled trials on TTPB.

This study has several limitations. First, because this study will be conducted at a single institution, it is possible that the patient background, anesthesia management, and surgical procedures may contain factors unique to our institution. Therefore, the external validity of the results of this study is limited, and generalization to other institutions or different patient populations requires careful interpretation. Second, this protocol was primarily aimed at investigating the effectiveness of TTPB on surgical site pain at the start of surgery. Thus, further randomized, controlled trials are needed regarding postoperative recovery, postoperative acute pain, and chronic pain. In addition, in this study, TTPB is to be performed before surgery, but the optimal timing for TTPB remains unknown. Based on the results of this study, future prospective, comparative studies are needed regarding the timing of TTPB, either before or after surgery, or both before and after surgery. Even taking these limitations into account, this study is considered significant given its contribution to improving perioperative analgesia, reducing opioid consumption, and avoiding local anesthetic toxicity. In addition, there have been no previous reports examining the effective concentration of local anesthetics used for ultrasound-guided nerve blocks at the start of cardiac surgery, and this study proposes a new approach.

## Conclusions

In summary, this study is to be a dose-finding study using a biased coin up-and-down method. To our knowledge, this is the first study aimed at determining the EC90 of TTPB under ultrasound guidance that will contribute to the growing body of evidence on TTPB. More broadly, this study will help promote multimodal pain management to spare opioid use in cardiac surgery.
